# Comparison of the Photoautotrophic Growth Regimens of *Chlorella sorokiniana* in a Photobioreactor for Enhanced Biomass Productivity

**DOI:** 10.3390/biology9070169

**Published:** 2020-07-16

**Authors:** Elvira E. Ziganshina, Svetlana S. Bulynina, Ayrat M. Ziganshin

**Affiliations:** Department of Microbiology, Institute of Fundamental Medicine and Biology, Kazan (Volga Region) Federal University, 420008 Kazan, Russia; elvira.ziganshina@kpfu.ru (E.E.Z.); SvSBulynina@stud.kpfu.ru (S.S.B.)

**Keywords:** *Chlorella sorokiniana*, PPFD, growth, nutrient removal, photobioreactor, productivity, characterization

## Abstract

Microalgae have a wide industrial potential because of their high metabolic diversity and plasticity. Selection of optimal cultivation methods is important to optimize multi-purpose microalgal biotechnologies. In this research, *Chlorella sorokiniana* AM-02 that was isolated from a freshwater lake was cultured under various high photosynthetic photon flux density (PPFD) conditions and CO_2_ gas levels in standard Bold’s basal medium (BBM). Furthermore, a wide range of nitrate levels (180–1440 mg L^−1^) was tested on the growth of *C. sorokiniana*. Microalgae growth, pigment concentration, medium pH, exit gas composition, as well as nitrate, phosphate, and sulfate levels were measured during an experimental period. The preferred high PPFD and optimal CO_2_ levels were found to be 1000–1400 μmol photons m^−2^ s^−1^ and 0.5–2.0% (*v*/*v*), respectively. The addition of nitrate ions (up to 1440 mg L^−1^) to the standard growth medium increased final optical density (OD_750_), cell count, pigment concentration, and total biomass yield but decreased the initial growth rate at high nitrate levels. Our findings can serve as the basis for a robust photoautotrophic cultivation system to maximize the productivity of large-scale microalgal cultures.

## 1. Introduction

Microalgae-based biotechnologies have received much attention worldwide, and they are presented as complex processes for purification of air and wastewater as well as for the production of substantial amounts of lipids, polysaccharides, proteins, commercially important pigments (such as lutein and astaxanthin), and other useful products. Pharmaceuticals for various purposes, nutraceuticals, fertilizers, biodiesel, bioethanol, and biogas are obtained from different microalgae species [1,2,3,4].

Currently, individual microalgae that are capable of adsorbing or transforming certain pollutants have become excellent candidate species for simultaneously capturing carbon dioxide and removing nutrients, hydrocarbons, pesticides, and cyanide compounds [1,5] as well as for remediation of aquatic ecosystems polluted with heavy metals [6]. The practical application of microalgae metabolic potential can be limited by certain factors, such as photoinhibition and a slow response to different light irradiance, which lead to a low yield of biomass or target products. To increase the cell growth rate, the efficiency of biomass production, and the yield of target products, which means the effectiveness of various microalgae-based technologies, light intensity, photoperiod, temperature, pH, adequacy of macro- and micronutrients, and cultivation regimen (autotrophy, heterotrophy, and mixotrophy) must be thoroughly studied [4,7,8,9].

Multifarious microalgae species, including algae of the genus *Chlorella* (*Chlorophyta*; *Trebouxiophyceae*; *Chlorellales*; *Chlorellaceae*), have been studied in various experimental projects to assess their potential for nutrient bioextraction, bioremediation, and bioenergy processes [7,10]. Species in the genus *Chlorella* are thought to have high potential for industrial use because of their fast and stable growth characteristics [11,12,13]. *Chlorella sorokiniana* is considered to be one of the promising *Chlorella* species that is used for wastewater treatments as well as for producing valuable products, including lipids, polysaccharides, proteins, and pigments [10,14,15,16]. *Chlorella sorokiniana* UTEX 1230 was originally isolated from a stream in Texas in 1951 by Dr. Constantine Sorokin [17], and more detailed information about this species of *Chlorella* was presented in subsequent research studies [18,19,20]. *C. sorokiniana* can grow effectively in a wide temperature range (25–40 °C) and under different light intensities [11,18,19,20].

Representatives of freshwater microalgae have the potential for large-scale cultivation. Researchers have developed various methods of cultivation depending on further practical application goals for the object that is being studied. In photoautotrophic regimens, microalgae cultures assimilate inorganic carbon, and light intensity can significantly affect CO_2_ assimilation and biomass production [3,10,21]. Among cultivation trends for various industrially attractive microalgae, attention to heterotrophic (substrate dependent) or mixotrophic cultivation (auto- and heterotrophic) is given, which explains the choice with a higher growth rate and lower operating costs of green microalgae [7,11,15]. Although microalgae cultivation under heterotrophic and mixotrophic conditions has some advantages, this process is at risk of contamination with heterotrophic microbes because organic compounds are used as carbon and energy sources [22], and this leads to the release of carbon dioxide as one of the major greenhouse gases. The use of gaseous CO_2_ as a carbon source by photoautotrophic microalgae contributes to sequestration of carbon dioxide, which is one way to reduce the risks that are associated with global warming [23,24]. Furthermore, when synthesis of the target products is the cell’s physiological response to high light stress, it is important to develop and apply effective strategies for photoautotrophic cultivation [10]. Therefore, additional studies are needed on the characteristics of algae metabolism at different light intensities and CO_2_ concentrations for each microalgae culture. Moreover, the isolation of new strains with high rates of nitrogen, phosphorus, and sulfur removal is important for the development of new wastewater treatment technologies.

Despite recent success in this research area, it is important to develop novel methods and optimize previous methods of growing and harvesting of microalgae so that many attractive species for biotechnology can be grown in a short time and with less corresponding energy use. *C. sorokiniana* as one of the attractive species for wastewater treatment was selected for the current study. Thus, this study evaluated the optimal high photosynthetic photon flux density (PPFD) conditions and CO_2_ levels on the growth rate and the ability to utilize important nutrients, such as nitrate, phosphate, and sulfate, by indigenous *C. sorokiniana* AM-02 that tolerates high irradiance. In addition, based on the optimal growth parameters for *C. sorokiniana*, the influence of additional nitrogen and sulfur sources on the biomass productivity was investigated. The results presented here offer a new strategy for improving productivity and decreasing resource costs.

## 2. Materials and Methods

### 2.1. Isolation and Identification of Microalgal Strain

The microalgae culture of *Chlorella sorokiniana* AM-02 was isolated from a freshwater lake located in Kazan (Russia) in 2018. The isolated strain was made axenic by continuous sub-culturing using Bold’s basal medium (BBM) [25] on agar plates supplemented with ampicillin and kanamycin (10 and 50 µg mL^−1^ in the medium, respectively). The axenic culture was identified using molecular analysis of the ribulose bisphosphate carboxylase large subunit (*rbcL*) gene and translational elongation factor Tu (*tufA*) gene markers. For this, a FastDNA spin kit (MP Biomedicals, Solon, OH, USA) and a FastPrep-24 homogenizer (MP Biomedicals, Solon, OH, USA) were used to extract DNA from microalgae in accordance with the manufacturer’s protocol. DNA was then quantified with a NanoDrop 2000 spectrophotometer (Thermo Fisher Scientific, Wilmington, DE, USA) and stored at −20 °C until use. Primers for the *rbcL* and the *tufA* genes were selected from published studies and were used to identify microalgae (Table 1). Amplicons obtained with these primers were purified using a QIAquick PCR Purification kit (Qiagen, Hilden, Germany) and sequenced on a 3730 DNA Analyzer (Thermo Fisher Scientific, Wilmington, DE, USA) according to the manufacturers’ protocols. Sequencing data was confirmed by BLAST searches in the GenBank’s database and using the BOLD Identification System for the ribulose bisphosphate carboxylase gene. The determined *rbcL* gene and *tufA* gene sequences were deposited in the GenBank database under accession numbers MT647139 and MT647140, respectively.

### 2.2. Cultivation Conditions

Before starting the experiments in a photobioreactor, *C. sorokiniana* was placed in 250 mL Erlenmeyer flasks containing 30 mL of BBM under sterile conditions to eliminate any contamination. The inoculum was grown with shaking at 120 rpm at +26 °C under continuous illumination (150 μmol photons m^−2^ s^−1^). After 5 days, the biomass was concentrated by centrifugation at 5000× *g* for 5 min and then used as an inoculum for experiments in a photobioreactor.

### 2.3. Cultivation in a Photobioreactor

In order to study the influence of operational factors on photoautotrophic cultivation of *C. sorokiniana* for accelerated growth and biomass production, microalga with an initial OD_750_ of 0.01 (optical density at 750 nm) was grown in a sterilized 3.6 L Labfors 4 Lux photobioreactor (Infors HT, Bottmingen, Switzerland) with a 2.4 L working volume with controlled luminous flux levels. The experiments were divided into three main groups: (1) cultivation under different photosynthetic photon flux density (PPFD) conditions, (2) cultivation at various levels of carbon dioxide, and (3) cultivation in the presence of various levels of nitrate and sulfate ions.

The photobioreactor was illuminated with 1–16 Gro-Lux tubes with high blue and red radiation (per tube: 120 lumen (lm), maximum: 1920 lm) uniformly distributed around the culture vessel. Light intensity was additionally measured using a photosynthetically active radiation meter (PAR meter, Apogee Instruments, Logan, UT, USA). In experiments on the selection of optimal lighting, we tested the growth of culture at different values of illumination. Three treatments were carried out under different PPFD conditions (1000, 1200, and 1400 μmol m^−2^ s^−1^). For an optimized strategy for the growth and the accumulation of biomass, we analyzed the following conditions of the carbon dioxide regimen: cultures sparged with atmospheric air only (~0.04% CO_2_) and cultures sparged simultaneously with atmospheric air and continuously added CO_2_ (finally 0.5%, 1.0%, and 2.0% CO_2_ (*v*/*v*)). Aeration (0.55 volume of air per volume of liquid per minute) was provided by a compressor via a 0.45 µm filter. Carbon dioxide addition was provided by thermal mass flow meter and controller (Vögtlin Instruments, Aesch, Switzerland). They were mixed and injected into the reactor. The temperature was controlled at +26–26.5 °C. A relatively low temperature was chosen in our experiments since high temperatures lead to higher control costs and higher energy consumption of the process. The reactor was continuously stirred at 120 rpm. In all experiments, light was maintained on a 16:8 light/dark cycle. When observing the foam, a sterile 2% *v*/*v* solution of antifoam (Antifoam B, Sigma-Aldrich, St. Louis, MO, USA) was added. The experiments continued in the range of 112–232 h. Light, temperature, medium pH, pressure inside the reactor, carbon dioxide flow, and the percentage of released oxygen and carbon dioxide were measured by the Infors devices and displayed online on a computer screen.

Samples for analysis of algal growth, cell number, pigment content, and ions concentration in the culture medium were collected every day. pH as well percentage of the released molecular oxygen and carbon dioxide were measured throughout the experiments. Two independent experiments were performed to test reproducibility, and the results are presented as the mean.

### 2.4. Measurement of Algal Growth and Biomass Dry Weight

Optical density was measured using a Lambda 35 spectrophotometer (Perkin Elmer, Singapore) at 750 nm. Cell suspensions were diluted prior to measurements to obtain a final OD_750_ of less than 0.5 for measurements. Cell number was additionally determined by using a hemocytometer. The determination was conducted in triplicate, and the mean values are presented along with standard deviations. After 112–232 h (depending on the experiment), the centrifuged biomass (at 5000× *g* for 10 min) was washed twice with sterile deionized water to remove medium salts and centrifuged again. The remaining microalgal pellets were dried at +80 °C for 48 h. The final biomass yield (g L^−1^) was calculated as the difference between the weight of a centrifuge tube with dried microalgal pellet and the weight of the biomass-free centrifuge tube.

### 2.5. Measurement of Pigment Concentrations

Chlorophylls a and b, carotenoids, and total pigments (mg L^−1^) were determined using dimethyl sulfoxide extraction and optical absorption correlation, as previously described by Chai et al. [8] and Wellburn [29]. Optical densities at 480, 649.1, and 665.1 nm were measured with a Lambda 35 spectrophotometer (Perkin Elmer, Singapore). The concentration of pigments was measured in triplicate, and the mean values are presented along with standard deviations.

### 2.6. Ion Chromatography

Nitrate, phosphate, and sulfate concentrations were measured using a Dionex ICS-900 Ion Chromatography System (Thermo Fisher Scientific, Wilmington, DE, USA) equipped with an IonPac AG22 (4 × 50 mm) guard column, and an IonPac AS22 (4 × 250 mm) analytical column. The mobile phase consisted of 4.5 mM Na_2_CO_3_/1.4 mM NaHCO_3_ at a flow rate of 1.5 mL min^−1^. Data acquisition and instrument control were performed with the Dionex Chromeleon software (Sunnyvale, CA, USA). Analyses were conducted in triplicate, and the mean values are presented with standard deviations.

### 2.7. Data Analysis 

The effects of PPFD, CO_2_, and nitrate concentration values on the photoautotrophic growth of *C. sorokiniana* were evaluated. The influence of these parameters on microalgal biomass was significant when the *p* value was below 0.05 (based on a two-tailed *t* test).

## 3. Results and Discussion

### 3.1. Effects of PPFD and CO_2_ Concentration Values on Growth of Algae

**Figure 1:**
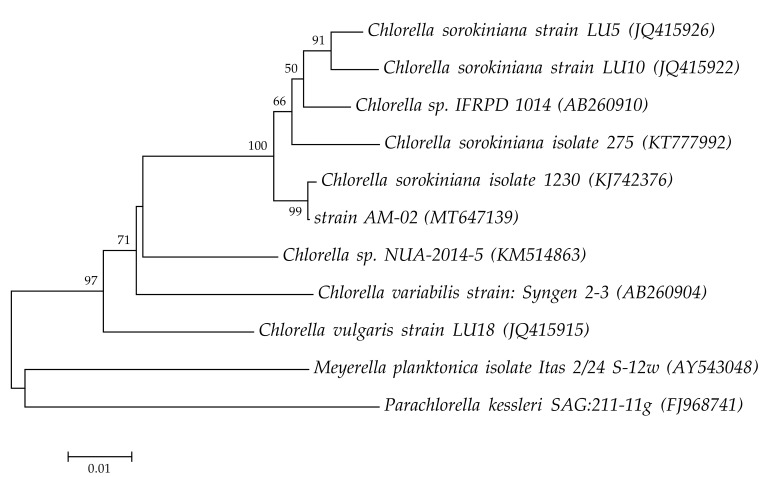
Phylogenetic tree based on *rbcL* gene sequences. Analysis was conducted in MEGA7 [30] using the neighbor-joining method. The percentages of replicate trees in which the associated taxa clustered together in the bootstrap test (1000 replicates) are shown next to the branches. The evolutionary distances were computed using the Kimura 2-parameter method. *Meyerella planktonica* and *Parachlorella kessleri* were used as outgroup.

In this research study, various PPFD and CO_2_ concentration values as well as nutrient requirements for maximum growth and biomass accumulation of *C. sorokiniana* AM-02 were determined. Phylogeny of the strain AM-02 based on *rbcL* gene is presented in Figure 1. Based on these data, this strain was assigned to *C. sorokiniana*.

**Figure 2:**
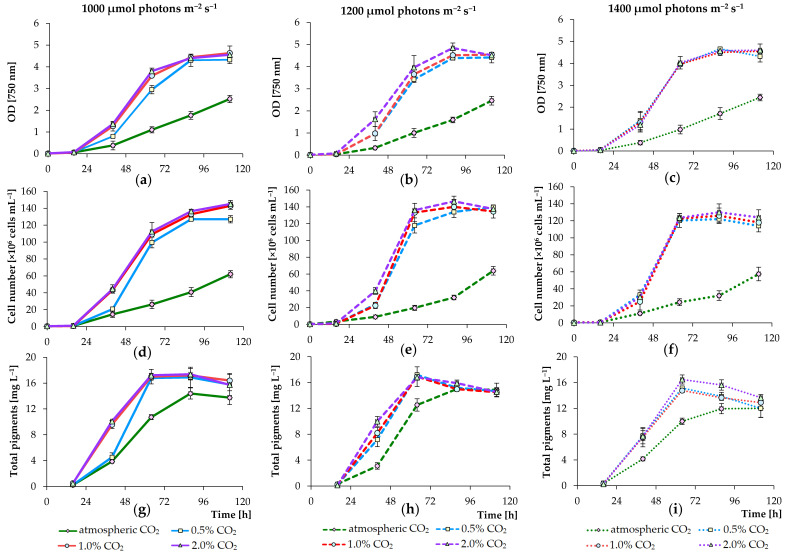
Growth of *C. sorokiniana* AM-02 (OD_750_ (**a**–**c**) and cell number (**d**–**f**)) and total pigment concentration in cells (**g**–**i**) cultured under various light and CO_2_ regimens (1000, 1200, and 1400 μmol photons m^−2^ s^−1^ and atmospheric CO_2_, 0.5, 1.0, and 2.0% CO_2_) in standard Bold’s basal medium (BBM).

To control the algal growth, OD_750_ was measured and number of cells was counted (results are summarized in Figure 2). Three high PPFD conditions were chosen (1000, 1200, and 1400 μmol m^−2^ s^−1^) for the experiments. We chose high PPFD conditions, because we plan to continue testing the growth of this strain in digested agricultural waste materials (such as pig manure and chicken manure), which are dark in color. In experiments, when cultures were bubbled with air supplemented with final 0.5%, 1.0%, and 2.0% CO_2_ (for all tested PPFD conditions), a 1.7–2.4-fold increase in growth in standard BBM was observed compared to that detected in treatments with non-CO_2_ augmented cultures. The mean OD_750_ values of *C. sorokiniana* grown at 1000, 1200, and 1400 μmol photons m^−2^ s^−1^ and sparged with air after 112 h of cultivation were 2.52, 2.46, and 2.44, respectively, whereas in experiments with the addition of extra CO_2_, the growth values were above 4.3 (Figure 2a–c).

The cultures in experiments supplied with different concentrations of carbon dioxide (under different PPFD conditions) increased in cell number until stationary growth was achieved at 1.22–1.46 × 10^8^ cells mL^−1^ (after 88 h), while in treatments supplied with atmospheric carbon dioxide, the cultures increased in cell number to 5.7–6.4 × 10^7^ cells mL^−1^ only (after 112 h) (Figure 2d–f). Overall, OD_750_ correlated with the calculated cell numbers (Figure 2). The concentration of total pigments (chlorophyll a, b, and total carotenoids) also increased to 14–17 mg mL^−1^ with higher accumulation in treatments supplemented with 2% carbon dioxide but decreased thereafter (Figure 2g–i).

Table 2 presents the tested regimens of cultivation and the average yield of dry biomass of microalgae after photoautotrophic cultivation. As can be seen from the cell density, cell number, average biomass yield of dry microalgae, as well as pigment content, the growth had the similar trends under all lighting conditions with an additional CO_2_ supply; however, the growth of *C. sorokiniana* was slightly higher in experiments in which PPFD and CO_2_ concentration values were 1200 μmol m^−2^ s^−1^ and 2.0%, respectively. In this regard, we used cultures grown at 1200 μmol photons m^−2^ s^−1^ and sparged with 2.0% carbon dioxide for further selection of optimal nutrient concentrations in modified BBM. At 1000 μmol photons m^−2^ s^−1^ and 0.5% CO_2_, the lower growth rate was additionally observed (in treatments with a changing level of CO_2_). However, at 1400 μmol photons m^−2^ s^−1^, the growth was almost identical regardless of the level of CO_2_ addition (0.5%, 1.0%, or 2%). It is believed that, from an industrial point of view, transportation, storage, and delivery of gaseous carbon dioxide are expensive for large-scale algae production [12]. Therefore, the cultivation of *C. sorokiniana* AM-02 on an industrial scale can also be achieved with lower levels of CO_2_ supplementation.

In several previous studies, other PPFD conditions (100–500 μmol photons m^−2^ s^−1^) were tested for culturing other *C. sorokiniana* strains in flasks and bottles, and no significant differences were found between different conditions for photoautotrophic cultures [6,11,13,31]. Due to the shading of the cells one above the other, the design of photobioreactors is also important to ensure adequate light distribution, as reported by Leon-Vaz et al. [32]. An increase in light intensity is not a solution to overcome shading in many photoautotrophic cultures with a high cell density, since many microalgae cannot grow efficiently at extremely high light intensities. However, Cuaresma et al. [33] reported a high growth rate of *C. sorokiniana* CCAP 211/8K under continuous illumination of 2100 μmol photons m^−2^ s^−1^ using red light emitting diodes. In our research, we show that our strain has optimal cultivation conditions in standard BBM at a high surface irradiance between 1000–1400 μmol photons m^−2^ s^−1^ with Gro-Lux tubes in a 3.6 L photobioreactor with a working volume of 2.4 L.

**Figure 3:**
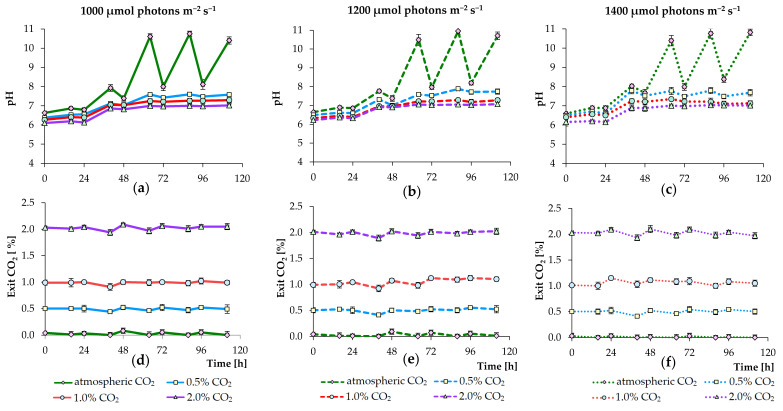
Changes in culture medium pH (**a**–**c**) and the released carbon dioxide level (**d**–**f**) observed during the growth of *C. sorokiniana* AM-02 cultured under various light and CO_2_ regimens (1000, 1200, and 1400 μmol photons m^−2^ s^−1^ and atmospheric CO_2_, 0.5, 1.0, and 2.0% CO_2_) in standard BBM.

Figure 3 demonstrates the changes in pH and the percentage of released CO_2_ under selected conditions. Thus, the pH increased from an initial ~6.6 to 10.4–10.9 under all lighting conditions in the non-CO_2_ augmented cultures (autotrophic cultivation leads to hydroxyl formation, which increases the pH), whereas the pH initially dropped to values 6.1–6.4 but ultimately did not increase above 7.8, 7.3, and 7.0 under all lighting conditions with the addition of CO_2_ up to 0.5%, 1.0%, and 2.0%, respectively (Figure 3a–c). During the light period, pH increased, while cultivation in the dark period led to a slight decrease in pH (in CO_2_ augmented cultures). In the non-CO_2_ augmented cultures, pH decreased substantially during the dark period. In the case of CO_2_ release from the photobioreactor, a decrease and an increase in the CO_2_ level were observed during the light period and the dark period under all lighting conditions, respectively (Figure 3d–f). The increase in pH found in the culture medium can be explained by the growth of *C. sorokiniana* and the uptake of carbon from dissolved HCO_3_^−^, leaving OH^–^ ions. Similarly, higher pH levels in the non-CO_2_ augmented cultures can be attributed to lower dissolved CO_2_ and less dissociated H^+^ ions [7,34]. In addition, sodium nitrate nutrition leads to an increase in pH of the medium [35].

**Figure 4:**
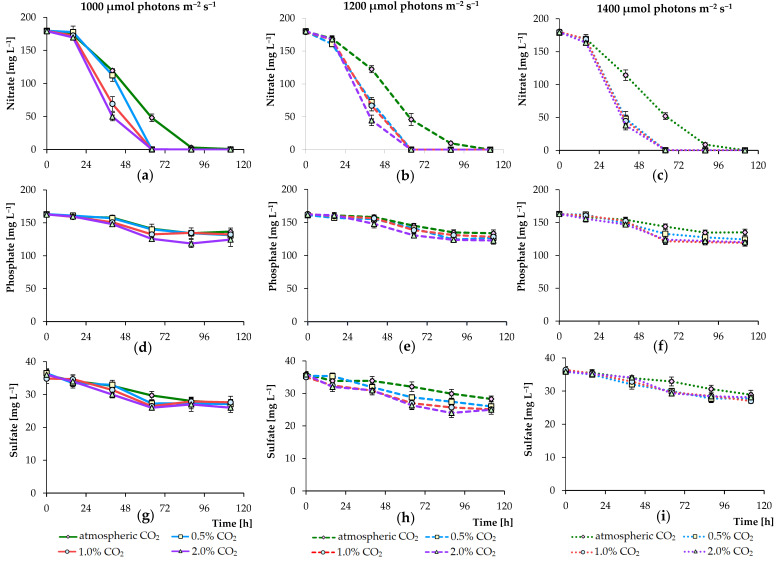
Changes in nitrate (**a**–**c**), phosphate (**d**–**f**), and sulfate (**g**–**i**) concentrations in culture medium during the growth of *C. sorokiniana* AM-02 cultured under various light and CO_2_ regimens (1000, 1200, and 1400 μmol photons m^−2^ s^−1^ and atmospheric CO_2_, 0.5, 1.0, and 2.0% CO_2_) in standard BBM.

Figure 4 shows the nutrient levels in the BBM with nitrate levels of about 180 mg L^−1^ at the beginning of cultivation. After 64 h and 88 h, nitrogen was completely utilized in standard BBM in the CO_2_ augmented and the non-CO_2_ augmented cultures, respectively, and, therefore, decreases in the growth of the microalgae cultures and the pigment concentration were noted. It should also be reported that nitrate, orthophosphate, and sulfate were consumed faster by culture of *C. sorokiniana* AM-02 when air containing 2.0% CO_2_ was continuously bubbled into the reactor. Ion chromatography analysis demonstrated that the added amounts of phosphate (around 160 mg L^−1^) and sulfate (around 35 mg L^−1^) slightly decreased but were in excess during the entire experimental period, while the nitrate concentration sharply decreased, and NO_3_^–^ completely was utilized after 64–88 h of cultivation (Figure 4). Thus, the concentration of NO_3_^–^ was initially insufficient for the active growth and development of the culture in photoautotrophic treatments; therefore, the growth values were low, and the following experiments were performed to identify the effects of increasing NO_3_^–^ content in the culture medium. These data confirm several reports in the literature on the rapid growth and rate of removal of nutrients but under other, different conditions [11]. In addition, some previous studies have demonstrated that various other *C. sorokiniana* strains can grow in wastewater with the effective removal of nitrates and phosphates [34,36], providing high potential for their application in developing wastewater treatment technologies.

### 3.2. Effects of Nutrient Concentration on Algal Growth

**Figure 5:**
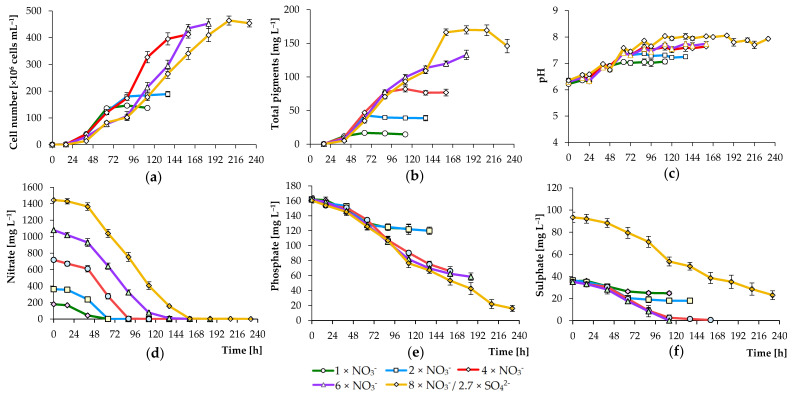
Growth of *C. sorokiniana* AM-02 (**a**), total pigment concentration in cells (**b**), culture medium pH (**c**), as well as nitrate (**d**), phosphate (**e**), and sulfate (**f**) removal from the medium in different treatments with modified BBM (PPFD and CO_2_ concentration values: 1200 μmol photons m^−2^ s^−1^ and 2.0% CO_2_, respectively).

In standard BBM (a medium that has been used to grow a variety of green algae cultures), NaNO_3_ is used as a source of nitrogen. The growth of strain AM-02 in treatments containing 180 mg L^−1^ NO_3_^–^ (standard concentration) under the selected PPFD and CO_2_ concentration values (1200 μmol m^−2^ s^−1^ and 2% CO_2_) was low, as measured by low cells number (1.47 × 10^8^ cells mL^−1^), pigment concentration (16.8 mg L^−1^), and dry biomass weight (1.10 g L^−1^) (Figure 5; Table 2). No NO_3_^–^ was detected in standard BBM sparged with air containing 2.0% CO_2_ on 66 h and onwards. Figure 5 shows that all key parameters were improved by adding an additional level of NO_3_^–^. In the 360 mg L^−1^ NO_3_^–^-treatments (double NO_3_^–^ concentration), number of cells, pigment concentration, and dry biomass weight increased significantly (*p <* 0.001; up to 1.9 × 10^8^ cells mL^−1^, 42.8 mg L^−1^, and 1.76 g L^−1^, respectively). However, even at a double concentration of introduced NO_3_^–^, nitrogen began to be limited after 66 h, and the cell concentration did not exceed 1.9 × 10^8^ cells mL^−1^. Under both conditions, PO_4_^3–^ and SO_4_^2–^ levels also decreased but remained abundant for culture growth (Figure 5).

A further increase in the concentration of the nitrogen source to 720 mg L^−1^ of NO_3_^–^ led to a significant increase in the number of cells and was reflected in the yield of final dry biomass (*p <* 0.001; Figure 5a; Table 2). After 160 h, the culture increased in cell concentration until reaching stationary growth at 4.13 × 10^8^ cells mL^−1^. After 112 h, the concentration of total pigments increased to 82.1 mg mL^−1^ and remained almost stable during cultivation. As shown in Figure 5b, the pigment concentrations in *C. sorokiniana* cultivated in the presence of high NO_3_^–^ levels were generally higher than those observed in cells cultured at lower levels of nitrate ions. A clear positive correlation was observed between chlorophyll levels and the color of the microalgae culture. High concentrations of NO_3_^–^ promoted the synthesis of pigments. Under these conditions, nitrate and sulfate were completely utilized by 88 h and 160 h, respectively (Figure 5d,f). Phosphate levels also decreased from an initial 160 to 66 mg L^−1^ by 160 h and remained at around these levels until the end of growth (Figure 5e). In 6×NO_3_^–^ and 8×NO_3_^–^-treatments, the final biomass yield and pigment concentration were significantly higher than previously reported (*p <* 0.001); however, the best growth rate was observed under 4×NO_3_^–^ conditions. Since SO_4_^2–^ was completely utilized under 4×NO_3_^–^ and 6×NO_3_^–^ conditions, we increased its level to 95 mg L^−1^ in 8×NO_3_^–^ treatments. Under all conditions, nitrate, phosphate, and sulfate were efficiently utilized by culture of *C. sorokiniana* (Figure 5). These results clearly demonstrate that *C. sorokiniana* AM-02 can grow at high nitrate concentrations and can be further tested for wastewater treatment. However, remarkably high NO_3_^–^ concentrations added to the medium reduced the specific growth, which can be explained by increased osmotic stress and inhibition by the intermediate [37].

The pH increased to 7.1, 7.3, 7.6, 7.8, and 8.0 in 1× NO_3_^–^, 2× NO_3_^–^, 4× NO_3_^–^, 6× NO_3_^–^, and 8× NO_3_^–^ -treatments, respectively (Figure 5c). In the case of CO_2_ emission from the photobioreactor, a decrease and an increase in the CO_2_ level were observed during the light period and the dark period, respectively, with an increase in the CO_2_ level by the entire period in experiments with a high nitrogen content (from an initial 2.0% to 2.6%, data not shown). Therefore, based on our results, nitrogen and sulfur are the main nutrients that become insufficient in standard BBM, while the phosphorous level is sufficient to support the growth of microalgae. Our findings also demonstrate that high level of inorganic anions removal is possible (100% for NO_3_^–^ and SO_4_^2–^ and 91% for PO_4_^3–^ (146 mg L^−1^)), which indicates the potential use of the strain for wastewater treatment.

Nutrient availability affects the growth of photoautotrophic organisms. One of the important components affecting the growth of microalgae and physiological activity is nitrogen [14,38,39]. For numerous applications of promising photosynthetic microorganisms, an increase in biomass productivity is considered to be an important step. Various potential nitrogen sources and their concentrations, such as urea, nitrate, and ammonium ions, have been evaluated by several other researchers for the cultivation of other *C. sorokiniana* strains. Lizzul et al. [34] observed that *C. sorokiniana* UTEX1230 prefers ammonium ions rather than nitrate ions as a source of nitrogen when cultured in wastewater and BBM medium. Ramanna et al. [36] reported that urea provided the highest biomass yield during cultivation of *C. sorokiniana* strain NIES: 2173 in wastewater. Kim et al. [7] found that growth rate and nitrogen and phosphorus removal rates by *C. sorokiniana* UTEX 1670 were higher under heterotrophic conditions than under autotrophic conditions. The wastewater nitrogen and phosphate were also effectively utilized by several other *C. sorokiniana* strains [40]. Another species of the same genus, *C. vulgaris*, also effectively removed nitrogen and phosphorus from domestic wastewater [41]. In this work, *C. sorokiniana* AM-02 was isolated from a freshwater lake and therefore can be adapted to different environmental changes and thus potentially provides a competitive advantage when grown in open water.

## 4. Conclusions

A study was conducted comparing the effect of various PPFD and CO_2_ concentration values on the growth of the new isolated strain *C. sorokiniana* strain AM-02. Thus, the photoautotrophic cultures of *C. sorokiniana* in modified BBM (720 mg L^−1^ of NO_3_^−^) showed better growth performance. It was found that the preferred high PPFD and CO_2_ concentration values are 1000–1400 μmol photons m^−2^ s^−1^ and 0.5–2.0%, respectively. In addition, the high growth of *C. sorokiniana* AM-02 makes this strain particularly suitable for the rapid production of biomass, attractive for bioremediation of aquatic environments contaminated with high levels of nitrate, phosphate, and sulfate. Moreover, our results show that adding an additional nitrogen source can significantly increase final biomass and pigment yield compared to standard BBM. Finally, our findings prove that the new isolated strain *C. sorokiniana* AM-02 is a fast-growing microalgae strain that can also be used in industry.

## Figures and Tables

**Table 1:** List of primers used in this study to identify the strain AM-02.

Molecular Marker	Primers	Sequence 5′ → 3′	Reference
*rbcL* (ribulose bisphosphate carboxylase large subunit)	*rbc*L-M379 F	GGTTTCAAAGCTYTWCGTGC	[26]
	*rbc*LFP R	GTAAATACCACGGCTACGRTCTT	
	Fw_rbcl_192	GGTACTTGGACAACWGTWTGGAC	[27]
	Rv_rbcL_657	GAAACGGTCTCKCCARCGCAT	
*tufA* (translational elongation factor Tu)	*tuf*A F	GGNGCNGCNCAAATGGAYGG	[28]
	*tuf*A R	CCTTCNCGAATMGCRAAWCGC	

**Table 2:** The average dry weight levels of algal biomass at the end of the experiments.

Regimen			Dry Weight (g L^−1^)
PPFD (μmol m^−2^ s^−1^)	Initial NO_3_^–^ (mg L^−1^)	Initial CO_2_ (%)	
1000	180	~0.04	0.43 ± 0.02
1000	180	0.5	1.07 ± 0.03
1000	180	1.0	1.08 ± 0.02
1000	180	2.0	1.02 ± 0.01
1200	180	~0.04	0.45 ± 0.02
1200	180	0.5	1.02 ± 0.02
1200	180	1.0	1.08 ± 0.04
1200	180	2.0	1.10 ± 0.03
1400	180	~0.04	0.44 ± 0.01
1400	180	0.5	1.05 ±0.02
1400	180	1.0	1.08 ±0.01
1400	180	2.0	1.07 ±0.02
1200	180	2.0	1.10 ± 0.03
1200	360	2.0	1.76 ± 0.08
1200	720	2.0	2.53 ± 0.12
1200	1080	2.0	3.11 ± 0.21
1200	1440	2.0	3.45 ± 0.24

PPFD: photosynthetic photon flux density.

## References

[1] Hammed A.M., Prajapati S.K., Simsek S., Simsek H. (2016). Growth regime and environmental remediation of microalgae. Algae.

[2] Chen B., Wan C., Mehmood M.A., Chang J.S., Bai F., Zhao X. (2017). Manipulating environmental stresses and stress tolerance of microalgae for enhanced production of lipids and value-added products—A review. Bioresour. Technol..

[3] Khan M.I., Shin J.H., Kim J.D. (2018). The promising future of microalgae: Current status, challenges, and optimization of a sustainable and renewable industry for biofuels, feed, and other products. Microb. Cell Fact..

[4] Saad M.G., Dosoky N.S., Zoromba M.S., Shafik H.M. (2019). Algal biofuels: Current status and key challenges. Energies.

[5] Wang L., Xiao H., He N., Sun D., Duan S. (2019). Biosorption and biodegradation of the environmental hormone nonylphenol by four marine microalgae. Sci. Rep..

[6] Kumar K.S., Dahms H., Won E., Lee J., Shin K. (2015). Microalgae—A promising tool for heavy metal remediation. Ecotox. Environ. Saf..

[7] Kim S., Park J.E., Cho Y.B., Hwang S.J. (2013). Growth rate, organic carbon and nutrient removal rates of *Chlorella sorokiniana* in autotrophic, heterotrophic and mixotrophic conditions. Bioresour. Technol..

[8] Chai S., Shi J., Huang T., Guo Y., Wei J., Guo M., Li L., Dou S., Liu L., Liu G. (2018). Characterization of *Chlorella sorokiniana* growth properties in monosaccharide-supplemented batch culture. PLoS ONE.

[9] Cecchin M., Benfatto S., Griggio F., Mori A., Cazzaniga S., Vitulo N., Delledonne M., Ballottari M. (2018). Molecular basis of autotrophic vs mixotrophic growth in *Chlorella sorokiniana*. Sci. Rep..

[10] Chen C.Y., Liu C.C. (2018). Optimization of lutein production with a two-stage mixotrophic cultivation system with *Chlorella sorokiniana* MB-1. Bioresour. Technol..

[11] Lizzul A.M., Lekuona-Amundarain A., Purton S., Campos L.C. (2018). Characterization of *Chlorella sorokiniana*, UTEX 1230. Biology.

[12] Lohman E.J., Gardner R.D., Pedersen T., Peyton B.M., Cooksey K.E., Gerlach R. (2015). Optimized inorganic carbon regime for enhanced growth and lipid accumulation in *Chlorella vulgaris*. Biotechnol. Biofuels.

[13] Bohutskyi P., Kligerman D.C., Byers N., Nasr L.K., Cua C., Chow S., Su C., Tang Y., Betenbaugh M.J., Bouwer E.J. (2016). Effects of inoculum size, light intensity, and dose of anaerobic digestion centrate on growth and productivity of *Chlorella* and *Scenedesmus* microalgae and their poly-culture in primary and secondary wastewater. Algal Res..

[14] Negi S., Barry A.N., Friedland N., Sudasinghe N., Subramanian S., Pieris S., Holguin F.O., Dungan B., Schaub T., Sayre R. (2016). Impact of nitrogen limitation on biomass, photosynthesis, and lipid accumulation in *Chlorella sorokiniana*. J. Appl. Phycol..

[15] Kobayashi N., Noel E.A., Barnes A., Watson A., Rosenberg J.N., Erickson G., Oyler G.A. (2013). Characterization of three *Chlorella sorokiniana* strains in anaerobic digested effluent from cattle manure. Bioresour. Technol..

[16] Lu S., Wang J., Niu Y., Yang J., Zhou J., Yuan Y. (2012). Metabolic profiling reveals growth related FAME productivity and quality of *Chlorella sorokiniana* with different inoculum sizes. Biotechnol. Bioeng..

[17] Sorokin C., Myers J. (1953). A high-temperature strain of *Chlorella*. Science.

[18] Sorokin C., Kraus R.W. (1959). Maximum growth rates of *Chlorella* in steady-state and in synchronized cultures. Proc. Nat. Acad. Sci. USA.

[19] Sorokin C., Krauss R.W. (1962). Effects of temperature and illuminance of *Chlorella* growth uncoupled from cell division. Plant Physiol..

[20] Sorokin C., Krauss R.W. (1965). The dependence of cell division in *Chlorella* on temperature and light intensity. Am. J. Bot..

[21] Min M., Hu B., Zhou W., Li Y., Chen P., Ruan R. (2012). Mutual influence of light and CO_2_ on carbon sequestration via cultivating mixotrophic alga *Auxenochlorella protothecoides* UMN280 in an organic carbon-rich wastewater. J. Appl. Phycol..

[22] Perez-Garcia O., Escalante F.M.E., de-Bashan L.E., Bashan Y. (2011). Heterotrophic cultures of microalgae: Metabolism and potential products. Water Res..

[23] Salih F.M. (2011). Microalgae tolerance to high concentrations of carbon dioxide: A review. J. Environ. Prot..

[24] Pavlik D., Zhong Y., Daiek C., Liao W., Morgan R., Clary W., Liu Y. (2017). Microalgae cultivation for carbon dioxide sequestration and protein production using a high-efficiency photobioreactor system. Algal Res..

[25] Nichols H.W., Bold H.C. (1965). *Trichosarcina polymorpha* Gen. et Sp. Nov. J. Phycol..

[26] Vieira H.H., Bagatini I.L., Guinart C.M., Vieira A.A.H. (2016). *tufA* gene as molecular marker for freshwater *Chlorophyceae*. Algae.

[27] Hadi S.I.I.A., Santana H., Brunale P.P.M., Gomes T.G., Oliveira M.D., Matthiensen A., Oliveira M.E.C., Silva F.C.P., Brasil B.S.A.F. (2016). DNA barcoding green microalgae isolated from Neotropical Inland waters. PLoS ONE.

[28] Fama P., Wysor B., Kooistra W.H.C.F., Zuccarello G.C. (2002). Molecular phylogeny of the genus *Caulerpa* (*Caulerpales*, *Chlorophyta*) inferred from chloroplast *tufA* gene. J. Phycol..

[29] Wellburn A.R. (1994). The spectral determination of chlorophylls a and b, as well as total carotenoids, using various solvents with spectrophotometers of different resolution. J. Plant Physiol..

[30] Kumar S., Stecher G., Tamura K. (2016). MEGA7: Molecular evolutionary genetics analysis version 7.0 for bigger datasets. Mol. Biol. Evol..

[31] Li T., Zheng Y., Yu L., Chen S. (2014). Mixotrophic cultivation of a *Chlorella sorokiniana* strain for enhanced biomass and lipid production. Biomass Bioenergy.

[32] León-Vaz A., León R., Díaz-Santos E., Vigara J., Raposo S. (2019). Using agro-industrial wastes for mixotrophic growth and lipids production by the green microalga *Chlorella sorokiniana*. New Biotechnol..

[33] Cuaresma M., Janssen M., Vílchez C., Wijffels R.H. (2009). Productivity of *Chlorella sorokiniana* in a short light-path (SLP) panel photobioreactor under high irradiance. Biotechnol. Bioeng..

[34] Lizzul A., Hellier P., Purton S., Baganz F., Ladommatos N., Campos L. (2014). Combined remediation and lipid production using *Chlorella sorokiniana* grown on wastewater and exhaust gases. Bioresour. Technol..

[35] Wang J., Zhou W., Chen H., Zhan J., He C., Wang Q. (2019). Ammonium nitrogen tolerant *Chlorella* strain screening and its damaging effects on photosynthesis. Front. Microbiol..

[36] Ramanna L., Guldhe A., Rawat I., Bux F. (2014). The optimization of biomass and lipid yields of *Chlorella sorokiniana* when using wastewater supplemented with different nitrogen sources. Bioresour. Technol..

[37] Gilmour D.J., Hipkins M.F., Boney A.D. (1984). The effect of osmotic and ionic stress on the primary processes of photosynthesis in *Dunaliella tertiolecta*. J. Exp. Bot..

[38] Ordog V., Stirk W.A., Balint P., Staden J., Lovasz C. (2012). Changes in lipid, protein and pigment concentrations in nitrogen-stressed *Chlorella minutissima* cultures. J. Appl. Phycol..

[39] Janssen J.H., Wijffels R.H., Barbosa M.J. (2019). Lipid production in *Nannochloropsis gaditana* during nitrogen starvation. Biology.

[40] Bohutskyi P., Liu K., Nasr L.K., Byers N., Rosenberg J.N., Oyler G.A., Betenbaugh M.J., Bouwer E.J. (2015). Bioprospecting of microalgae for integrated biomass production and phytoremediation of unsterilized wastewater and anaerobic digestion centrate. Appl. Microbiol. Biotechnol..

[41] Mayhead E., Silkina A., Llewellyn C.A., Fuentes-Grünewald C. (2018). Comparing nutrient removal from membrane filtered and unfiltered domestic wastewater using *Chlorella vulgaris*. Biology.

